# Diagnosis and Treatment of Adenocarcinomas and Squamous Cell Carcinomas of the Lung

**Published:** 2017-04-01

**Authors:** Millie Das, Alison Holmes Tisch

**Affiliations:** 1 Stanford University and VA Palo Alto Health Care System;; 2 Stanford Cancer Center

## Abstract

Following years in which there were only modest gains in treatment options for non-small cell lung cancer, recognition of targetable mutations and immunogenicity of lung cancer now impact treatment decisions.

In the treatment of advanced lung cancer, histology still guides treatment selection, and a wealth of new targeted and immunotherapeutic agents is changing the natural history of this challenging malignancy, according to two Stanford University clinicians who described the management of adenocarcinomas and squamous cell carcinomas of the lung at JADPRO Live 2016.

Millie Das, MD, of Stanford University and VA Palo Alto Health Care System, summarized the emerging treatment landscape, and Alison Holmes Tisch, MSN, RN, ANP-BC, AOCNP®, of Stanford Cancer Center, described the workup and illustrated the management challenges through case reports. Together, they summarized the key elements in management: involving molecular testing, matching mutations to targeted agents, initiating immunotherapy at the right time and in the right patients, and ameliorating toxicities.

## PATIENT PRESENTATION AND WORKUP

"The diagnosis of lung cancer can be challenging, as symptoms can be vague at first and are often attributed to other illnesses," Ms. Tisch said. Typical local symptoms are cough, dyspnea, hemoptysis, and chest pain. General symptoms often include fatigue and weight loss. Secondary to distant metastasis are bone pain and neurologic symptoms. The rare patient may develop paraneoplastic syndromes.

Initial imaging is often a chest radiograph, but a more complete workup involves chest computed tomography (CT), including the adrenals, a common metastatic site. Positron-emission tomography (PET) and dedicated brain magnetic resonance imaging (MRI) can reveal distant metastases. Since molecular testing requires adequate tissue sampling, most patients undergo bronchoscopy, CT-guided biopsy, or thoracentesis, she said.

"With the biopsy, we are hoping to establish the diagnosis, determine the histologic subtype, and perform molecular testing. Make sure to get a decent-sized cell block. Ideally, we like to do a core biopsy," advised Ms. Tisch.

The first distinction in diagnosis is between small cell (10%–15%) and non–small cell (85%–90%) lung cancer (NSCLC). Within NSCLC, adenocarcinoma is most common (30%–40%), followed by small cell (20%–25%), with large cell (10%–15%) and other/mixed types (3%–5%) being less common (American Cancer Society, 2017). It is important to understand the distinctions between adenocarcinoma and squamous histologies (Table), Dr. Das said.

**Table 1:**
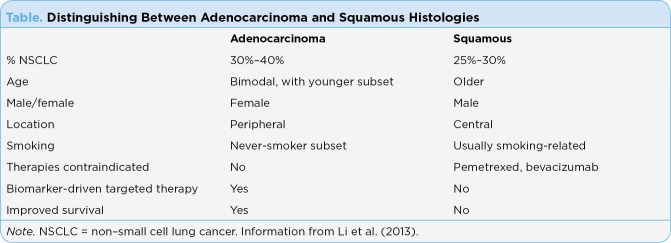
Distinguishing Between Adenocarcinoma and Squamous Histologies

Molecular testing is now an important part of the workup, based on the understanding of driver mutations and the availability of drugs that target them.

In brief, patients with adenocarcinoma, large cell cancer, and NSCLC not-otherwise-specified should be tested for mutations in the epidermal growth factor receptor (*EGFR*) and translocations of anaplastic lymphoma kinase (*ALK*) as part of a broader molecular profiling; if these factors are negative or unknown, patients should be tested for ROS1 rearrangement. Patients with squamous cell tumors are candidates for molecular testing if they are never-smokers or have a mixed histology. Mutations of *EGFR* are present in about 50% of never-smokers and in about one-third of light smokers (D’Angelo et al., 2011), according to Dr. Das.

Increasingly, other genes are being evaluated, including *KRAS*, *BRAF*, *HER2*, and *RET*. "We are increasingly interested in next-generation sequencing to look for other potential targets," Dr. Das said. Although drugs targeting these additional mutations are not yet available, knowledge of the genetic landscape "helps us understand what’s driving their cancer, and there may be off-label or clinical trial options for them," Ms. Tisch added.

The recent approval of pembrolizumab (Keytruda), an antibody against PD-1, in the first-line setting adds another testing option at diagnosis. To receive this immune checkpoint inhibitor (for patients lacking targetable mutations), patients must demonstrate ≥ 50% expression of the programmed cell death ligand 1 (PD-L1), Ms. Tisch added.

Rebiopsy at the time of disease progression, especially in patients with mutations, helps determine the mechanisms of resistance and informs decisions regarding second-line options. In some patients, it is possible to do so via "liquid biopsy."

## SELECTING CHEMOTHERAPY REGIMEN

Treatment selection for stage IV NSCLC is based on histologic subtype, mutation status, patient performance status, smoking status, presence of central nervous system (CNS) disease, and receipt of prior systemic therapy in the adjuvant or locally advanced setting. The main drug categories are chemotherapy (choice depends on histologic subtype), targeted agents for patients with mutations (especially *EGFR*, *ALK*, and *ROS1*), and immune checkpoint inhibitors (anti–PD-1/PD-L1 and antibodies against cytotoxic T-lymphocyte–associated protein 4 [anti–CTLA-4]).

The standard first-line chemotherapy for stage IV NSCLC is a platinum doublet given for four cycles. The landmark Eastern Cooperative Oncology Group (ECOG) 1594 trial established this as the treatment of choice (Schiller et al., 2002). Overall survival was essentially the same for the four regimens—cisplatin/paclitaxel, cisplatin/gemcitabine, cisplatin/docetaxel, and carboplatin/paclitaxel. But with better tolerability than cisplatin, the doublet of carboplatin/paclitaxel became the standard of care, Dr. Das said.

This study was followed by an important phase III noninferiority trial by Scagliotti et al. (2008), who showed that, for some drugs, histology matters. In the first-line setting, cisplatin/pemetrexed (Alimta) improved survival over cisplatin/gemcitabine in patients with adenocarcinoma or large cell NSCLC, whereas cisplatin/gemcitabine was preferred in patients with squamous cell tumors. In patients with nonsquamous disease, median overall survival was 11.8 months in the pemetrexed arm, vs. 10.4 in the gemcitabine arm, leading to the approval of pemetrexed for advanced-stage disease with nonsquamous histology.

Following this study in patients with nonsquamous histology, ECOG 4599 demonstrated a 2-month survival benefit with the addition of bevacizumab (Avastin) to carboplatin/paclitaxel in the first-line setting (Sandler et al., 2006). The use of bevacizumab remains restricted to the nonsquamous subset, as patients with squamous tumors had excess mortality related to pulmonary hemorrhage.

In squamous histology, nanoparticle albumin-bound (nab)-paclitaxel (Abraxane; plus carboplatin) almost doubled the response rate (41%) over paclitaxel/carboplatin (24%) in a large front-line trial (Socinski et al., 2012), although overall survival in the entire population (squamous and nonsquamous) was similar (11–12 months). Also in squamous cell patients, necitumumab (Portrazza), a second-generation antibody targeting EGFR, when added to cisplatin/gemcitabine, led to an overall survival benefit of 1.6 months in the phase III SQUIRE front-line trial (Thatcher et al., 2015), leading to this drug’s approval.

## MAINTENANCE AND SECOND-LINE CHEMOTHERAPY

"A number of studies are evaluating the role of maintenance therapy after patients complete first-line therapy with a platinum doublet," Dr. Das said. There are several options: (1) continuous maintenance, where the patient completes four to six cycles of a platinum doublet (with or without bevacizumab); if the patient achieves at least stable disease, he or she continues to receive one or two of the same agents; (2) switch maintenance, in which the patient with stable disease or response then switches to pemetrexed, docetaxel, or erlotinib (Tarceva; assuming the patient did not receive this drug in the first-line setting); (3) "early" second-line therapy, where drugs are offered that might delay progression; this option might be used, for example, for the patient who could not receive a certain drug in the first-line setting due to disease burden.

"While the data support maintenance, many patients don’t want to continue chemotherapy after they are done," acknowledged Dr. Das. "It’s not the right thing for all patients."

In the second-line setting, chemotherapy could include the immunoglobulin G (IgG) antibody targeting the vascular endothelial growth factor receptor 2 (VEGFR-2) ramucirumab (Cyramza). In the phase III REVEL trial, the addition of ramucirumab to docetaxel improved overall survival by 1.4 months, leading to its US Food and Drug Administration (FDA) approval in 2015 (Garon et al., 2014).

## TARGETED THERAPY

For patients with advanced NSCLC and driver mutations, first-line treatment includes a targeted agent. Mutations in *EGFR* occur in 10% to 15% of all patients with NSCLC and predict responsiveness to the EGFR tyrosine kinase inhibitors (TKIs) erlotinib, gefitinib (Iressa), and afatinib (Gilotrif). Mutations of *EGFR*, which occur most often in EGFR exons 18–21, are mostly associated with never-smokers, those with adenocarcinoma histology, females, and persons of Asian ethnicity.

The landmark IPASS trial proved the benefit of an EGFR TKI—gefitinib—in patients with *EGFR*-mutated cancers (Mok et al., 2009). The LUX-Lung trials evaluated another EGFR TKI, afatinib, showing clear benefit over chemotherapy in *EGFR*-mutated patients (Sequist et al., 2013, 2014; Wu et al., 2014). Patients with exon 19 deletion had the most pronounced survival benefit with afatinib: 33.3 months vs. 21.1 months for chemotherapy (hazard ratio [HR] = 0.54; p = .002) in LUX-Lung 3 (Sequist et al., 2014).

"This added to the data we have supporting EGFR TKI treatment for mutation-positive patients in the front-line setting," she said. According to the IMPRESS study of gefitinib (Soria et al., 2015), there is no benefit to continuing an EGFR TKI beyond disease progression.

But as effective as the EGFR TKIs are, they are not curative, as most patients become resistant after about 1 year of treatment. It is now standard to look for resistance mutations (especially EGFR T790M, seen in 60% of patients) in recurrent tumors, blood, or urine.

"It is important to identify the specific *EGFR* mutation. There are sensitive mutations, primary resistance mutations, and acquired resistance mutations, especially T790M. For a patient with primary resistance, for example, you might not necessarily offer an EGFR TKI first line," Dr. Das explained. The IMPASS trial showed the detriment of treating non–*EGFR*-mutated patients with an EGFR TKI (Mok et al., 2009). "This changed the way we practice. In mutation-negative patients, we give platinum doublets upfront," she emphasized.

Identifying the T790M mutation has become especially important now that a third-generation EGFR TKI, osimertinib (Tagrisso), has been shown to be effective in this setting and is now approved. In a study by Jänne et al. (2015), 61% of T790M-positive patients responded to osimertinib, whereas 21% of T790M-negative patients responded.

Dr. Das acknowledged that although toxicities with EGFR TKIs are usually mild, the occurrence of acneiform rash and diarrhea require intervention in about 30% of patients. Proactive management can reduce their severity and maximize treatment outcomes.

## *ALK* AND *ROS* REARRANGEMENTS

Rearrangements in the EML4-ALK fusion protein are seen in about 5% of patients with NSCLC adenocarcinoma. They are most common in younger male patients who are light- or never-smokers, and they are believed to be mutually exclusive of *EGFR* mutations, according to Dr. Das.

Front-line treatment for *ALK*-positive patients is an ALK inhibitor. In a phase I study, 60.8% of such patients responded to crizotinib (Xalkori; Camidge et al., 2012). The clear benefits observed in this study led to a change in practice: to initiate treatment with an ALK inhibitor in these patients. Even more potent is ceritinib (Zykadia), which produced robust responses as well in a phase I study (Shaw et al., 2014a). Its activity is independent of prior ALK-inhibitor therapy, "making this a great option for patients treated with crizotinib upfront who develop resistance," Dr. Das indicated.

The most recently approved ALK inhibitor is alectinib (Alecensa). In two pivotal studies (Ou et al., 2016; Shaw et al., 2016), alectinib produced responses in approximately 50% of patients, including those with prior chemotherapy and CNS metastases. "Its activity in CNS disease (response rates of 57% and 75%) is higher than we’ve seen with crizotinib or even ceritinib," she revealed. "Though alectinib’s current indication is for crizotinib-refractory patients, there is interest in using it in the front-line setting, especially in patients with significant CNS metastases."

Crizotinib is also a first FDA-approved choice for patients with *ROS1* fusions, based on a response rate of 72% in this population, a median duration of response of 17.6 months, and a median progression-free survival of 19.2 months (Shaw et al., 2014b).

Other genomic targets are emerging in lung cancer, including *BRAF*, *RET* fusion, and *MET* exon 14 splice mutation. In early trials, these subsets are responding to dabrafenib (Tafinlar) with and without trametinib (Mekinist), cabozantinib (Cometriq), and cabozantinib plus crizotinib, respectively. "With every day that passes, we are finding more targets, and companies are developing active drugs against them," Dr. Das commented.

## IMMUNOTHERAPY

"The hot new topic in oncology is immunotherapy," Dr. Das said. The PD-1/PD-L1 inhibitors have gained an established role now in NSCLC, with the following approvals:

- Nivolumab (Opdivo; PD-1 inhibitor) for squamous and nonsquamous NSCLC in the second-line setting and beyond. No testing for PD-L1 expression is required, and patients lacking PD-L1 expression can receive nivolumab.
- Pembrolizumab (PD-1 inhibitor) for squamous and nonsquamous NSCLC in both the first-line and second-line settings. In the first-line setting, patients must have ≥ 50% expression of PD-L1 on the companion diagnostic assay (PD-L1 IHC 22C3 pharmDx test); in the second-line setting, expression of PD-L1 ≥ 1% is required.
- Atezolizumab (Tecentriq; PD-L1 inhibitor) is approved in the second-line setting; PD-L1 testing is not required, and patients lacking PD-L1 expression can receive the drug.

The approval of nivolumab for patients with squamous cell disease was based on the results of CheckMate 017, which showed a median overall survival of 9.2 months with nivolumab and 6.0 months with docetaxel (HR = 0.59; *p* < .001), in patients progressing after one platinum doublet (Brahmer et al., 2015). In patients with nonsquamous disease, approval was also granted, based on 1-year survival of 51% with nivolumab vs. 39% with docetaxel in CheckMate 057 (Borghaei et al., 2015). Recently, the recommendation was made to give nivolumab at a fixed dose of 240 mg intravenously every 2 weeks (rather than 3 mg/kg), Dr. Das said.

In 2015, pembrolizumab was approved for patients with NSLC progressing on a platinum doublet, based on KEYNOTE-001. Although initially approved in the second-line setting for patients with ≥ 50% PD-L1 expression, the indication was recently revised to allow its use in patients with ≥ 1% PD-L1 expression.

The biggest evolution in this field came with the results of KEYNOTE-024 (Reck et al., 2016). In previously untreated patients with ≥ 50% PD-L1 expression, median progression-free survival was 10.3 months with pembrolizumab vs. 6.0 months with a platinum doublet (HR = 0.50; *p* < .001), and median overall survival at 6 months was 80.2% vs. 72.4% (HR = 0.60; *p* = .005).

"With these exciting results, we got the first approval of a checkpoint inhibitor front line," Dr. Das noted.

The anti–PD-L1 antibody atezolizumab was also recently approved for second-line use and beyond. In the POPLAR trial, median overall survival was 12.6 months with atezolizumab vs. 9.7 months with docetaxel (HR = 0.69; Barlesi et al., 2016).

Dr. Das noted that immune checkpoint inhibitors have a unique toxicity profile, related to autoimmunity, which clinicians must learn to manage. This topic was covered in depth by other speakers at JADPRO Live 2016.

"This is clearly a very exciting time in the treatment of lung cancer, with many recent drug approvals," Dr. Das concluded. "The ultimate goal will be to individualize treatment for patients based on specific biomarkers and genetic alterations, leading to treatments that, hopefully, will improve survival."
